# Prediction of Synaptically Localized RNAs in Human Neurons Using Developmental Brain Gene Expression Data

**DOI:** 10.3390/genes13081488

**Published:** 2022-08-20

**Authors:** Anqi Wei, Liangjiang Wang

**Affiliations:** 1 Department of Genetics and Biochemistry, Clemson University, Clemson, SC 29634, USA; 2 Center for Human Genetics, Clemson University, Greenwood, SC 29646, USA

**Keywords:** RNA localization, synapses, neurons, machine learning, expression features

## Abstract

In the nervous system, synapses are special and pervasive structures between axonal and dendritic terminals, which facilitate electrical and chemical communications among neurons. Extensive studies have been conducted in mice and rats to explore the RNA pool at synapses and investigate RNA transport, local protein synthesis, and synaptic plasticity. However, owing to the experimental difficulties of studying human synaptic transcriptomes, the full pool of human synaptic RNAs remains largely unclear. We developed a new machine learning method, called PredSynRNA, to predict the synaptic localization of human RNAs. Training instances of dendritically localized RNAs were compiled from previous rodent studies, overcoming the shortage of empirical instances of human synaptic RNAs. Using RNA sequence and gene expression data as features, various models with different learning algorithms were constructed and evaluated. Strikingly, the models using the developmental brain gene expression features achieved superior performance for predicting synaptically localized RNAs. We examined the relevant expression features learned by PredSynRNA and used an independent test dataset to further validate the model performance. PredSynRNA models were then applied to the prediction and prioritization of candidate RNAs localized to human synapses, providing valuable targets for experimental investigations into neuronal mechanisms and brain disorders.

## 1. Introduction

How people memorize, learn, and process external information largely depends on the sophisticated connections between neurons [1]. Unlike typical cells, neurons have a highly polarized architecture, consisting of the soma with the nucleus, and extended protrusions, including dendrites and an axon [2]. Within a complex neural network, the region where two neurons contact is referred to as a synapse, which is essential for neural communications [3]. Extensive studies have been conducted to understand mRNA transport and localization to synapses. It is commonly acknowledged that many mRNAs are packaged into granules after being transcribed in the nucleus and then transported to synaptic regions for local translation. The mechanism of local translation is supposed to facilitate fast responses to environmental changes and synaptic inputs [4,5]. Thus, mRNA localization plays a key role in neuronal protein translation, allowing the local synthesis of components required for synaptic plasticity during brain development [5,6,7,8]. Dysregulation of synaptic mRNA localization and translation can affect cellular functions, leading to neurological diseases such as Fragile X Syndrome and Spinal Muscular Atrophy [9,10]. Moreover, synaptically localized RNAs may be involved in liquid–liquid phase separation to form membraneless neurite compartments with diverse functions [11].

With highly polarized morphology, neurons offer a great model for studying RNA localization [8]. Subcellular fractionation techniques and electron microscopy were originally used to understand the structure of synaptic terminals and internal contents [12,13,14]. Recently, microarrays [2,6,15] and next-generation sequencing technologies have been used to profile dendritic transcriptomes in rats and mice [16,17,18,19,20]. However, it is very challenging to accurately profile dendritic transcriptomes, with major difficulties in the clean separation of dendrites from cell bodies and the complexity of dynamic neuropil events [18]. Notably, previous studies have only identified a small number of dendritic RNAs in common [16,17,18,19,20], and the full synaptic RNA pool remains largely unclear.

Previous studies suggest that neuronal mRNAs may carry regulatory elements that affect mRNA localization, stability, and translation [19,20], whereas the lack of localization signals could be the reason why some mRNAs are retained in the soma [21]. For instance, the 3′ UTR of Ca^2+^/calmodulin-dependent protein kinase II (*CaMKIIa*) targets the mRNA to dendrites for local translation [22]. The loss of localization signals in the 3′ UTR of *CaMKIIa* mRNA altered its distribution in dendrites, resulting in reduced accumulation of *CaMKIIa* in postsynaptic densities (PSD) and impairments of synaptic plasticity and spatial memory [23]. The 5′ UTR of sensorin mRNA has also been implicated in synaptic mRNA localization in *Aplysia* [7]. These findings suggest that sequence features may be used to predict synaptic RNAs.

Since many neuronal proteins are involved in synaptic plasticity and higher-order brain functions such as learning and memory [24], synaptic genes may also display characteristic expression patterns during neuronal development and aging. Interestingly, the analysis of human brain time-series transcriptome data reveals that synaptic genes are particularly sensitive to the aging process [25]. Moreover, functional genomic studies using developmental human brain transcriptome data have shown that schizophrenia and autism spectrum disorders partially converged on neurodevelopmental modules involved in transcriptional regulation and synaptic function [26,27]. Thus, gene expression data may also contain relevant information for predicting synaptic RNAs.

With the growing size and complexity of genomic data, machine learning techniques have been increasingly used to extract hidden knowledge regarding a specific biological problem. One intriguing problem is RNA subcellular localization, which plays an important role in modulating protein distributions and cellular functions of various classes of RNAs transcribed from the genome [28]. To date, machine learning models have been developed to predict the subcellular localization of RNAs, with some models intended for mRNAs [29,30,31,32] and others for long non-coding RNAs (lncRNAs) [33,34,35]. RNATracker used a deep neural network to predict the subcellular localization of mRNAs from one-hot-encoded transcript sequences [31]. mRNALoc employed support vector machine (SVM) models to predict mRNA subcellular localization based on pseudo-K-tuple nucleotide composition (PseKNC) features [29]. Recently, DM3Loc was developed, which applied the multi-head self-attention mechanism to deep learning architecture [32]. For lncRNAs, predictors such as lncLocator [33], iLoc-lncRNA [34] and DeepLncRNA [35] utilized sequence-based features. lncLocator and iLoc-lncRNA were constructed using conventional machine learning algorithms, and DeepLncRNA was based on a deep neural network. Many methods mentioned above enable multi-label prediction for multiple subcellular regions such as the nucleus, cytoplasm, ribosome, exosome, and so on. However, no model has yet been reported to our knowledge for accurate prediction of synaptically localized RNAs.

In this study, we developed a new machine learning method named PredSynRNA to predict human synaptically localized RNAs. We compiled a training dataset from previous studies and used RNA sequence and developmental brain gene expression data as features to construct various models with different learning algorithms. Interestingly, the Support Vector Machine (SVM) model using the expression features achieved the best performance. PredSynRNA was then employed to predict and prioritize candidate RNAs, including 1070 mRNAs and 330 lncRNAs, which might be localized to human synapses.

## 2. Materials and Methods

### 2.1. Compilation of Training Data Instances

Considering the lack of training instances of human dendritic and somatic RNAs, we first collected those from respective lists published in five rodent studies that utilized RNA-sequencing techniques [16,17,18,19,20]. For each RNA instance, we identified the human orthologue using Ensembl BioMart [36]. To improve the quality of the dataset, we examined the overlaps across different studies and only selected the dendritic and somatic RNAs identified by at least two independent studies as potential instances using jvenn [37] (Figure S1). In addition, any instances that overlapped with potential training positives were excluded from the list of somatic RNAs. The dataset before feature encoding contained 1423 dendritically localized RNAs (positive instances) and 1617 somatically localized RNAs (negative instances). Most, if not all, of the dendritic RNAs were considered to be synaptically localized as axonal RNAs were normally at very low abundance when synaptic transcriptomes were profiled in the previous studies [16,17,18,19,20].

### 2.2. Sequence and Expression Features

It was suggested that certain sequence elements might be responsible for mRNA localization to synaptic neuropil [7]. We thus extracted sequence features by calculating the *k*-mer frequencies of concatenated 5′ and 3′ UTR of an mRNA transcript (normalized by the sequence length). Protein-coding transcript sequences were downloaded from the GENCODE GRCh38 release 33 [38], and the longest protein-coding sequence was retained. Sequence features derived from different *k*-mer combinations (*k* = 1, 2, 3) were examined for model construction (Figure S2).

The gene expression features for each RNA instance were extracted from the BrainSpan Atlas of the Developing Human Brain [39]. The BrainSpan dataset contained the expression profiles of over 52,000 genes in 524 brain tissue samples from 26 brain structures for a series of developmental time points ranging from 8 weeks post-conception (pcw) to 40 years of age. The gene expression levels were represented by Reads Per Kilobase of transcript per Million mapped reads (RPKM). The RNA instances with RPKM > 1 in at least 1% of brain samples were retained, resulting in a training dataset of 1271 positive instances and 1513 negative instances. The expression features were processed by log_2_(RPKM + 1) transformation. The expression and sequence features were also normalized using the min–max method.

### 2.3. Feature Selection

The high dimensionality of sequence and expression features might lead to model overfitting. Feature selection could be utilized to remove redundant and irrelevant features [40]. It was also of interest to identify and examine the most important features for predicting synaptically localized RNAs. During model training, the importance score of each feature was computed using the Random Forest (RF) algorithm [41]. The mean importance scores calculated from five repetitions of 10-fold cross-validations were used to rank and select the most relevant features. The importance scores of expression features were also examined to reveal the significant time points during brain development.

### 2.4. Model Training

Various machine learning algorithms, including logistic regression (LR), support vector machine (SVM), random forest (RF), XGBoost (XGB), and artificial neural network (ANN), were tested for model construction. LR is a statistical method that finds the best fitting model to describe the relationship between the logit of outcome and a set of independent variables. SVM is a learning algorithm that aims to distinguish two classes by a hyperplane with the maximal margin [42]. RF is an ensemble learning method that constructs a multitude of decision trees for a classification task [41]. XGBoost is an implementation of gradient-boosted decision trees and has fast execution speed and good model performance [43]. In this study, the LR, SVM, and RF models were implemented using Scikit-learn 0.21.2 [44] and XGB with xgboost 0.90. To find the optimal set of parameters for each model, the grid search method was used. The class weights within the parameters were set for the above models to address the imbalance of the training dataset. For the ANN model, different numbers of hidden layers were tested, and the ANN with one hidden layer was chosen in this study (Tables S1 and S2). The optimization of hyperparameters, including hidden units, drop-out rate, and learning rate, was performed using Hyperopt 0.2.4 [45]. The ANN model was implemented with Keras 2.2.4 in Python. Tuned parameters for three final, most representative models used for future analysis are provided in Table S3.

### 2.5. Model Testing

During model construction, PredSynRNA performance was evaluated by five repetitions of 10-fold cross-validations, in which the training dataset was randomly divided into 10 equal-sized subsets: one holdout subset for testing and the remaining nine subsets for training [46]. For the ANN model, an additional step of bootstrap resampling was used to obtain a balanced dataset before 10-fold cross-validations.

Furthermore, an independent test dataset was collected from a previous study on the somato-dendritic localization of mRNAs in mouse hippocampus [47] and used to validate the generalization ability of PredSynRNA. Any instances in the training dataset were excluded from the independent test dataset. Sequence and expression features were extracted in the same way as for the training instances. This test dataset contained 613 positive instances and 925 negative instances.

### 2.6. Performance Metrics

The performance metrics used in this study are as follows:

(1) $$\mathrm{Accuracy}\;=\frac{\mathrm{TP}+\mathrm{TN}}{\mathrm{TP}+\mathrm{TN}+\mathrm{FP}+\mathrm{FN}},$$

(2) $$\mathrm{Sensitivity}=\frac{\mathrm{TP}}{\mathrm{TP}+\mathrm{FN}},$$

(3) $$\mathrm{Specificity}=\frac{\mathrm{TN}}{\mathrm{TN}+\mathrm{FP}},$$

(4) $$F1=2\times \frac{\mathrm{Precision}\;\times \;\mathrm{Recall}}{\mathrm{Precision}+\mathrm{Recall}},$$

(5) $$\mathrm{MCC}=\frac{\mathrm{TP}\times \mathrm{TN}-\mathrm{FP}\times \mathrm{FN}}{\sqrt{\left(\mathrm{TP}+\mathrm{FP}\right)\times \left(\mathrm{TP}+\mathrm{FN}\right)\times \left(\mathrm{TN}+\mathrm{FP}\right)\times \left(\mathrm{TN}+\mathrm{FN}\right)}}.$$

True positives (TP), true negatives (TN), false positives (FP), and false negatives (FN) are tabulated and used to calculate the performance metrics shown above. The Matthews correlation coefficient (MCC) measures the correlation between the predicted and actual classifications on a scale of 0 ≤|MCC| ≤ 1 [48]. The receiver operating characteristic (ROC) curve plots the true positive rate (sensitivity) versus the false positive rate (1–specificity) for varying output thresholds of the model. The ROC curve and the area under the curve (ROC-AUC) are considered the most robust measures of model performance [49].

### 2.7. Prediction and Analysis of Candidate RNAs Localized to Human Synapses

After model validation, PredSynRNA was applied to the prediction of synaptically localized candidate RNAs from a list of brain-expressed RNAs, including 7046 mRNAs and 3331 lncRNAs. The top three PredSynRNA models with the best performance in cross-validations and on the independent test dataset were used to predict the probability of a given RNA transcript being synaptically localized, with the default probability threshold of 0.5. The positive predictions shared by all the three models were referred to as the high-confidence list of candidate RNAs.

To understand the biological processes or cellular functions in which the high-confidence candidates might be involved, we performed functional annotation clustering analysis using DAVID Bioinformatics Resources 6.8 with the list of brain-expressed genes as the background [50]. High classification stringency was used, and the EASE score referring to the one-tail Fisher exact probability value for the enrichment analysis was set to 0.01.

The high-confidence list of candidate RNAs was also compared with the SynGO gene list, which included 1112 synaptic genes based on gene ontology (GO) annotations and published, expert-curated evidence [51]. GSEAPreranked analysis (GSEA 4.1.0) with default parameters [52] was performed to examine the enrichment of SynGO genes in the ranked list of the brain expressed RNAs according to the probability scores predicted by PredSynRNA.

## 3. Results

**Figure 1:**
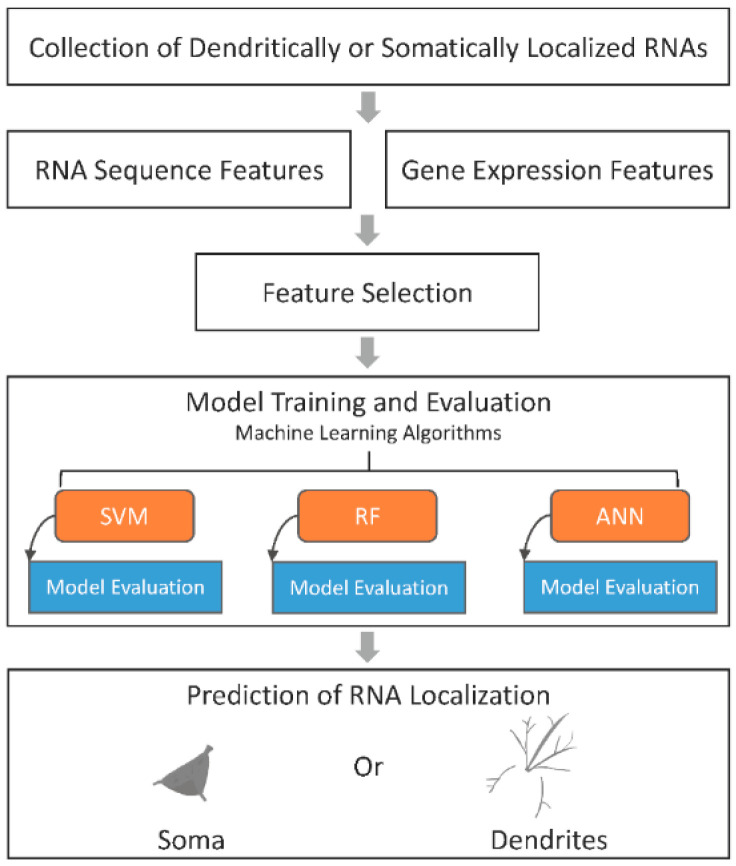
Schematic diagram of PredSynRNA for prediction of synaptically localized RNAs. First, dendritically and somatically localized RNAs were compiled from previous rodent studies. Then, human orthologues were identified and taken as the training instances. Second, features were extracted from RNA sequence and developmental brain gene expression data. Third, feature selection was conducted using a Random Forest-based method. Forth, various machine learning models were constructed and evaluated. Lastly, the best models were applied to the prediction and prioritization of candidate RNAs that may be localized to human synapses.

The machine learning task in this study can be defined as a binary classification problem, and our method, PredSynRNA, is illustrated in Figure 1. Dendritically and somatically localized RNAs were compiled from previous rodent studies [16,17,18,19,20] (Figure S1) due to the lack of published RNA instances in human neurons. Human orthologues were identified, and the RNAs shared in at least two studies were selected and taken as training instances. For feature encoding, the *k*-mer frequencies of RNA transcript sequences and the developmental brain gene expression profiles from the BrainSpan Atlas of the Developing Human Brain [39] were used to construct models with different learning algorithms. A Random Forest-based method was used for feature selection, and model performance was evaluated by 10-fold cross-validations and an independent test dataset. The best models were then utilized to predict and prioritize synaptically localized candidate RNAs.

### 3.1. Prediction of Synaptically Localized RNAs Using Sequence and Expression Features

**Figure 2:**
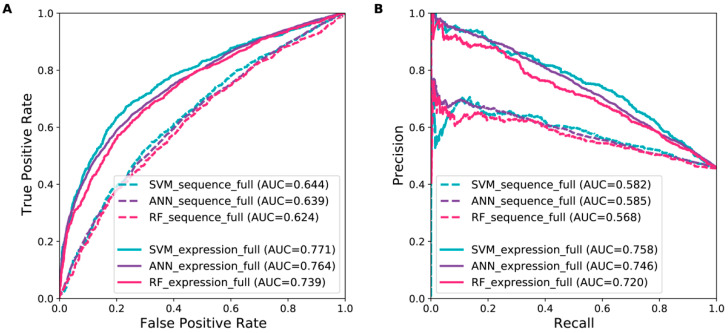
ROC (**A**) and PR (**B**) curves of different machine learning models using sequence and expression features. Model performance is based on five repetitions of 10-fold cross-validations, and the area under the curve (AUC) for each model is given in the legend.

We first constructed and evaluated various machine learning models using sequence features in terms of *k*-mer frequencies. Figure 2 show the ROC and precision-recall (PR) curves of the SVM, ANN, and RF models using a combination of 1-mer, 2-mer, and 3-mer frequencies. Since the training dataset was imbalanced, PR curves were used to show the model’s ability to predict positive instances [53]. A full comparison of the models using different sequence features is shown in Figure S2. The SVM model appeared to slightly outperform the ANN and RF models and achieved the ROC-AUC of 0.644 and PR-AUC of 0.582 (Figure 2 and Table 1). Although the different machine learning models using sequence features did not show good performance, they achieved higher ROC-AUC values than random guesses (ROC-AUC = 0.5), suggesting that the 5′ and 3′ UTRs might contain some relevant information for predicting synaptically localized RNAs.

Next, we built different machine learning models using developmental brain gene expression features. Based on the ROC and PR curves from 10-fold cross-validations, the expression-based models clearly outperformed the sequence-based models (Figure 2 and Figure S3). Particularly, the expression-based SVM model achieved the ROC-AUC of 0.771 and PR-AUC of 0.758, considerably higher than those of the sequence-based SVM model (Figure 2, Table 1 and Table S4). The results suggest that developmental brain gene expression profiles contain highly relevant information for predicting synaptically localized RNAs. However, model performance was not further improved by combining the expression features with the inherently different sequence features (Table S4).

### 3.2. Relevant Expression Features Learned by PredSynRNA

Feature selection was performed in this study to potentially improve model performance and to identify the most relevant features for predicting synaptically localized RNAs. We first computed the importance score of each feature using the RF-based method and then utilized the top-ranked expression or sequence features to build various machine learning models. However, when compared with using the full feature sets, feature selection did not significantly improve the performance of the expression or sequence-based models (Figures S4 and S5; Tables S4 and S5). For the expression features, as the dimensionality increased to 192 features, the models with different learning algorithms appeared to reach close to the maximum performance (Figure S4), suggesting that the top-ranked expression features captured most of the relevant information for predicting synaptically localized RNAs.

**Figure 3:**
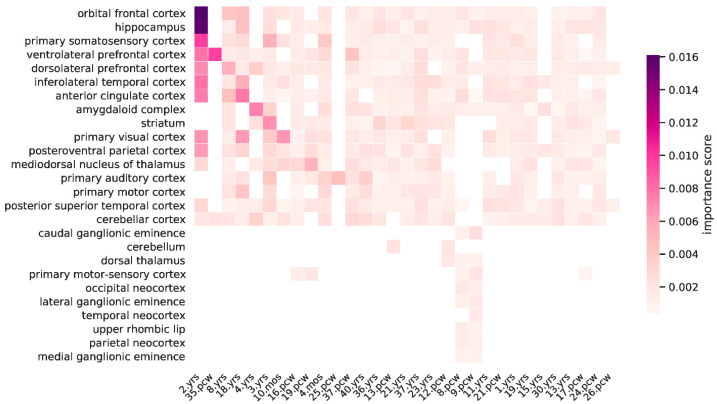
Visualization of the RF-based importance scores for the developmental brain gene expression features. The expression features with no importance score were excluded, and the importance scores of expression features in the same developmental time point and tissue type were averaged. The labels on the x-axis and y-axis have been arranged in descending orders based on the importance ranks of developmental time points and brain tissue types, respectively (pcw: post conception week; mos: months; yrs: years).

We examined the expression features, which included a series of developmental time points and brain tissue types of the samples in the BrainSpan dataset. As shown in Figure 3, the top three developmental time points based on the importance scores of the expression features included 2 years, 35 post-conception weeks, and 8 years, whereas the top three brain tissue types were found to be the orbital frontal cortex (OFC), hippocampus (HIP), and primary somatosensory cortex (S1C). The OFC is a prefrontal cortex region, which is critical in many aspects of brain function, including cognitive abilities, decision making, emotional processing, semantic memory, and language [54,55]. The HIP plays a key role in memory, learning, and spatial orientation [56]. The S1C is part of the somatosensory system, which is known for processing various somatosensory inputs from the body and has recently been shown to be involved in emotional regulation [57]. Taken together, our findings from feature selection are generally consistent with the knowledge that an explosion of synaptogenesis occurs in cortical regions during early brain development [58,59], further suggesting that the PredSynRNA models have learned relevant expression features for predicting synaptically localized RNAs.

### 3.3. Evaluation of Model Performance on an Independent Test Dataset

**Figure 4:**
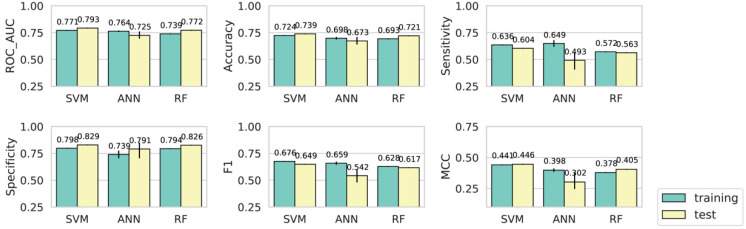
Validation of model performance using an independent test dataset. The SVM, ANN, and RF models were constructed using the full set of developmental brain gene expression features. Model performance metrics in cross-validations (training) and on the independent test dataset (test) with error bars are shown.

To further evaluate the predictive performance of the models, we compiled an independent test dataset with 613 positive instances and 925 negative instances, which were not included in the training dataset. Notably, almost all the tested models achieved comparable performance on the independent test dataset as in cross-validations (Figure 4 and Table S5). The performance metrics of the SVM, ANN, and RF models using the full expression features are depicted in Figure 4. Interestingly, when compared with model performance in cross-validations, the SVM and RF models achieved slightly higher ROC-AUC, accuracy, and MCC on the independent test dataset, whereas the ANN model showed slightly reduced performance, probably due to the fact that ANN could be easily overfitted on a small training dataset and the model generalization ability might be affected. In addition, feature selection did not improve model performance on the independent test dataset (Table S5). Overall, the results confirmed the predictive capability of the PredSynRNA models using developmental brain gene expression data.

### 3.4. Prediction and Prioritization of Candidate Human RNAs Localized to Synapses

To identify synaptically localized candidate RNAs, we applied the SVM, ANN, and RF models trained with the full expression features to classify a list of 10,377 brain-expressed RNAs, including 7046 mRNAs and 3331 lncRNAs. Overall, 2747, 1348, and 2777 mRNAs were predicted to be synaptically localized mRNAs by the SVM, ANN, and RF models, respectively (Figure S6 and Table S6). Particularly, 1070 candidate mRNAs were shared by the three lists of predictions. Moreover, 330 lncRNAs were commonly predicted by the three PredSynRNA models (Figure S7 and Table S7). These common predictions were regarded as high-confidence candidate RNAs that may be localized to human synapses.

**Figure 5:**
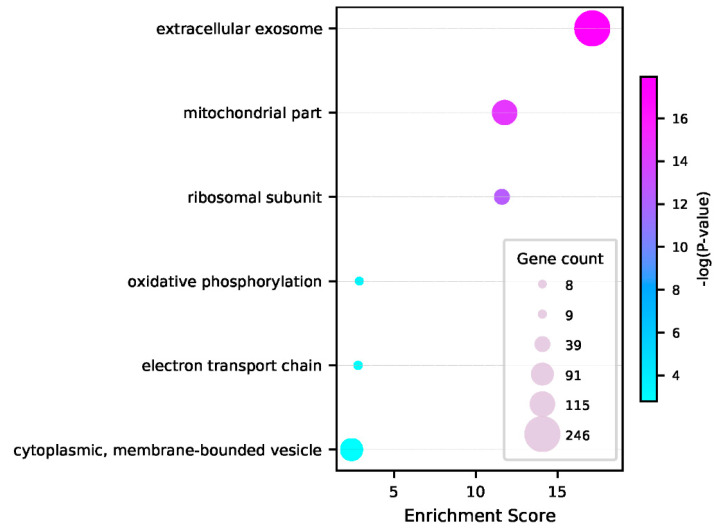
Functional terms enriched within the high-confidence candidate RNAs. The DAVID functional annotation clustering analysis was performed for the list of 1400 high-confidence candidate RNAs predicted by PredSynRNA to be synaptically localized. GO terms (GOTERM_BP_4, GOTERM_CC_4, and GOTERM_MF_4) were used for the functional analysis. For each annotation cluster, the most enriched GO term, its gene count, and statistical significance are shown in the diagram.

To characterize the high-confidence candidates, we performed DAVID functional annotation clustering analysis [50]. As shown in Figure 5, six functional terms were found to be significantly enriched in the candidate list, including extracellular exosome, mitochondrial part, and ribosomal subunit as the top three gene ontology (GO) terms. Exosomes, a class of extracellular vesicles, have been shown to play key roles in the central nervous system, synaptic plasticity, and inter-neuronal communication [60,61]. At the synapse, membrane-bound vesicles store neurotransmitters, enabling the transfer of information between neuron cells [62]. In addition, neurons highly rely on aerobic oxidative phosphorylation together with the principal energy producers, mitochondria, to support synapse dynamics. The dysfunctions of these crucial factors may contribute to the pathology associated with neurodegenerative disorders such as Alzheimer’s disease [63,64]. Moreover, differential expression analysis in a previous study [18] suggested that the mitochondrial membrane, ribosomal subunit, and electron transport chain are among the top GO terms enriched in dendrites. Therefore, the results demonstrated a significant association between the candidate RNAs and synapse-related functions.

**Figure 6:**
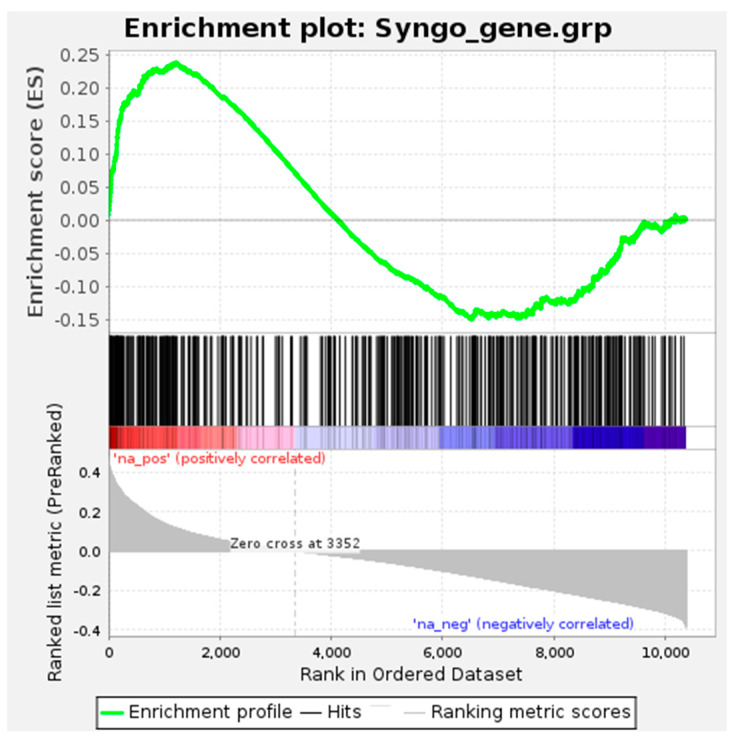
Significant enrichment of synaptic genes in the ranked list of candidate RNAs. The list of 10,377 brain-expressed RNAs was ranked by the mean probability scores predicted by the SVM, ANN, and RF models using the full set of expression features. The GSEAPreranked analysis [51] was then performed for a set of 1112 human synaptic genes obtained from the SynGO database [50]. The enrichment score (ES) reaches the maximum (0.2373) near the top of the ranked list, and the nominal *p*-value is estimated to be zero by an empirical phenotype-based permutation test procedure (actual *p*-value < 0.001 with 1000 permutations).

To further examine the functional association with synapses, we compared the candidate RNAs with a set of 1112 human synaptic genes curated by the SynGO database [51]. The list of 10,377 brain-expressed RNAs was ranked by the mean probability scores predicted by the SVM, ANN, and RF models of PredSynRNA, and the enrichment of synaptic genes in the ranked list was analyzed using the GSEAPreranked algorithm [52]. As shown in Figure 6, the synaptic genes from SynGO are significantly enriched near the top of the ranked list, where the candidate RNAs are located. The enrichment score (ES) reaches the maximum (0.2373) near the top of the ranked list, and the nominal *p*-value is estimated to be zero (actual *p*-value < 0.001). A list of 82 SynGO synaptic genes showing core enrichment is provided in Table S8. Taken together, our results suggest that the PredSynRNA models can be used to prioritize the candidate RNAs for investigating their functional roles in human synapses.

## 4. Discussion

RNA localization to synapses is not only regarded as one of the driving forces for developmental changes in the brain but is also implicated in neurological diseases. While machine learning methods have been developed for predicting RNA localization to multiple cellular compartments [29,30,31,32,33,34,35], such predictors are still lacking for synaptically localized RNAs. In this study, we developed a new machine learning method, PredSynRNA, to predict the synaptic localization of human RNAs. PredSynRNA models utilized developmental brain gene expression data as features and achieved relatively high performance in cross-validations and on an independent test dataset. Our results also suggest that the models can capture relevant expression features for predicting and prioritizing candidate RNAs localized to human synapses. However, the performance of PredSynRNA might be limited due to the lack of experimentally verified human RNA instances for model training. To construct the models, we used human orthologues of rodent RNAs identified by previous studies, which had only a small number of dendritic RNAs in common. Thus, PredSynRNA model performance may be further improved by compiling a more comprehensive and high-quality training dataset for this difficult machine learning task in the future.

Despite the limited and noisy training data, PredSynRNA models using the developmental brain gene expression features achieved relatively high performance for predicting synaptically localized RNAs. However, the addition of RNA sequence features in terms of *k*-mer frequencies did not further improve model performance. This is rather surprising as many previous studies attempted to identify potential localization elements present in the untranslated regions, mostly the 3′ UTRs of mRNA transcripts in neurites. Since these elements can be heterogeneous to a great extent in size and structure, it may be hard to predict and deduce the consensus sequence or structural motifs [65]. Moreover, mRNA localization in neurites can also be affected by alternative splicing and polyadenylation. Previous studies have also shown that neuronal mRNAs are prone to have diverse 3′ UTR isoforms, which differ in subcellular locations, including soma and neurites [19,20,66,67]. Therefore, simple sequence features such as *k*-mer frequencies may not be able to delineate the complex patterns of RNA localization to synapses.

Nevertheless, the results do not necessarily mean that RNA transcript sequences do not contain relevant information for predicting synaptically localized RNAs. In future studies, state-of-the-art deep learning techniques may be utilized to uncover the sequence patterns that determine RNA localization to synapses. It is noteworthy that deep learning techniques have been used to identify sequence motifs for mRNA subcellular localization to the nucleus, cytosol, endoplasmic reticulum, and exosome [31,32]. RNATracker [31] implemented a convolutional neural network (CNN) coupled with bi-directional long short-term memory (LSTM) layers to learn and extract sequence information for predicting mRNA subcellular localization, and the weights learned by the first CNN layer were converted into position–weight matrices and matched with known motifs of RNA-binding proteins to reveal the localization zip codes. DM3Loc [32] employed multiscale CNN filters and multi-head self-attention layers to infer the localization zip codes. However, the lack of high-quality localization data and the complexity of alternative splice variants for synaptically localized RNAs make it difficult to apply sophisticated deep learning techniques.

Since the robust performance of PredSynRNA was demonstrated by cross-validations and using an independent test dataset, the models were then utilized to predict and prioritize candidate RNAs, mostly mRNAs, which might be localized to human synapses. Interestingly, the top five candidate mRNAs include *RPL8*, *MZT2B*, *RPS20*, *TMEM219*, and *HBB* (Table S6). RPL8 has been identified as one of the candidate proteins that are significantly associated with the prognosis of the most aggressive brain cancer-glioblastoma and temozolomide treatment [68]. *MZT2B* has been reported to be one of the potential hippocampus genes associated with Alzheimer’s disease [69]. *RPS20* has been suggested as a candidate gene associated with medulloblastoma, the most common malignant brain tumor in children [70]. The *TMEM219* gene is located in a multigenetic copy number variation region (16p11.2) associated with several brain disorders, including schizophrenia, seizure, and Alzheimer’s disease [71,72,73]. HBB has been shown to be in mitochondrial fractions of mammalian neurons and involved in neuronal metabolism to provide neuroprotection in multiple sclerosis [74,75,76,77]. The PredSynRNA models have also been used to predict a list of synaptically localized candidate lncRNAs, including *SNHG8* and *MALAT1* (Table S7). As the full set of human synaptic RNAs remains largely unclear, we anticipate that the high-confidence candidate RNAs predicted by PredSynRNA can provide valuable targets for further experimental studies. However, it should be noted that the human brain is the most complex organ, which comprises different cell types of great diversity [78]. Although PredSynRNA has been trained using the developmental brain gene expression data with most samples derived from cortex regions that tend to have high neuronal enrichment, the predicted candidate RNAs may also be expressed in other non-neuronal cell types such as glial cells. With the accumulation of single-cell RNA-seq data, which provide fine resolution in examining cellular compositions and dynamics during brain development [78,79], PredSynRNA may be further refined by incorporating comprehensive, high-quality cell-type specific data in the future.

## 5. Conclusions

In this study, we developed a new machine learning method, PredSynRNA, to predict the synaptic localization of human RNAs. The PredSynRNA model utilized developmental brain gene expression data as features to achieve relatively high performance during cross-validations and on an independent test dataset. Our results also suggest that the model is capable of capturing relevant expression features and can be used to predict and prioritize candidate RNAs localized to human synapses. In the future, PredSynRNA model performance may be further improved by compiling and curating a more comprehensive and high-quality training dataset for this difficult machine learning task.

## Figures and Tables

**Table 1:** Performance metrics of models using different features and learning algorithms. Support vector machine (SVM), artificial neural network (ANN), and random forest (RF) models achieved better performance using the expression features than the sequence features based on five repetitions of 10-fold cross-validations.

Features	Model	ROC-AUC	Accuracy	Sensitivity	Specificity	F1	MCC
Sequence_full	SVM	0.644	0.615	0.529	0.688	0.556	0.220
	ANN	0.639	0.603	0.549	0.649	0.554	0.201
	RF	0.624	0.597	0.523	0.660	0.542	0.184
Expression_full	SVM	0.771	0.724	0.636	0.798	0.676	0.441
	ANN	0.764	0.698	0.649	0.739	0.659	0.398
	RF	0.739	0.693	0.572	0.794	0.628	0.378
Expression_Sequence_full	SVM	0.768	0.722	0.639	0.791	0.676	0.436

## Data Availability

All the datasets, source code, and supplementary data are available at https://github.com/BioDataLearning/PredSynRNA (accessed on 18 August 2022) [80].

## Supplementary Materials

The following supporting information can be downloaded at: https://www.mdpi.com/article/10.3390/genes13081488/s1, Figure S1: Venn diagrams to show the overlaps of (A) dendritic RNAs and (B) somatic RNAs across five previous studies; Figure S2: ROC-AUCs of the LR, SVM, RF, XGB, and ANN models with various k-mer features based on (A) five repetitions of 10-fold cross-validations and (B) an independent test dataset; Figure S3: ROC and PR curves of the SVM, ANN, RF, LR, and XGB models with either sequence or expression features based on five repetitions of 10-fold cross-validations; Figure S4: ROC-AUCs of the models with selected expression features based on (A) five repetitions of 10-fold cross-validations and (B) the independent test dataset; Figure S5: ROC-AUCs of the models with the selected sequence features based on (A) five repetitions of 10-fold cross-validations and (B) the independent test dataset; Figure S6: Venn diagram of mRNAs predicted to be synaptically localized by the SVM, ANN, and RF models using the full set of developmental brain gene expression features; Figure S7: Venn diagram of lncRNAs predicted to be synaptically localized by the SVM, ANN, and RF models using the full set of developmental brain gene expression features; Table S1: Performance comparison of artificial neural networks (ANNs) with different numbers of hidden layers based on five repetitions of 10-fold cross-validations; Table S2: Performance comparison of artificial neural networks (ANNs) with different numbers of hidden layers based on five repetitions using the independent test dataset; Table S3: Training parameters tuned for the SVM, ANN, and RF models of PredSynRNA; Table S4: Performance comparison of the models with different feature sets based on five repetitions of 10-fold cross-validations; Table S5: Performance comparison of the models with different feature sets based on five repetitions using the independent test dataset; Table S6: List of high-confidence candidate mRNAs predicted by PredSynRNA using the developmental brain gene expression features from BrainSpan; Table S7: List of high-confidence candidate lncRNAs predicted by PredSynRNA using the developmental brain gene expression features from BrainSpan; Table S8: List of SynGO synaptic genes that show core enrichment in the GSEAPreranked analysis.
