# Supplementary material for: Prediction of Synaptically Localized RNAs in Human Neurons Using Developmental Brain Gene Expression Data
Source: Genes (Basel). 2022 Aug 20;13(8):1488. doi: 10.3390/genes13081488 (PMC9408096; doi:10.3390/genes13081488)
Supplement: Supplementary file 1 [file genes-13-01488-s001.zip › Supporting_Information.pdf]

Supporting Information

Prediction of synaptically localized RNAs in human neurons using developmental brain gene expression data

Anqi Wei and Liangjiang Wang*

Department of Genetics and Biochemistry, and Center for Human Genetics, Clemson University, Clemson, SC 29634, USA

*To whom correspondence should be addressed. Tel: 1-864-656-0733; Fax: 1-864-656-0393; Email: liangjw@clemson.edu

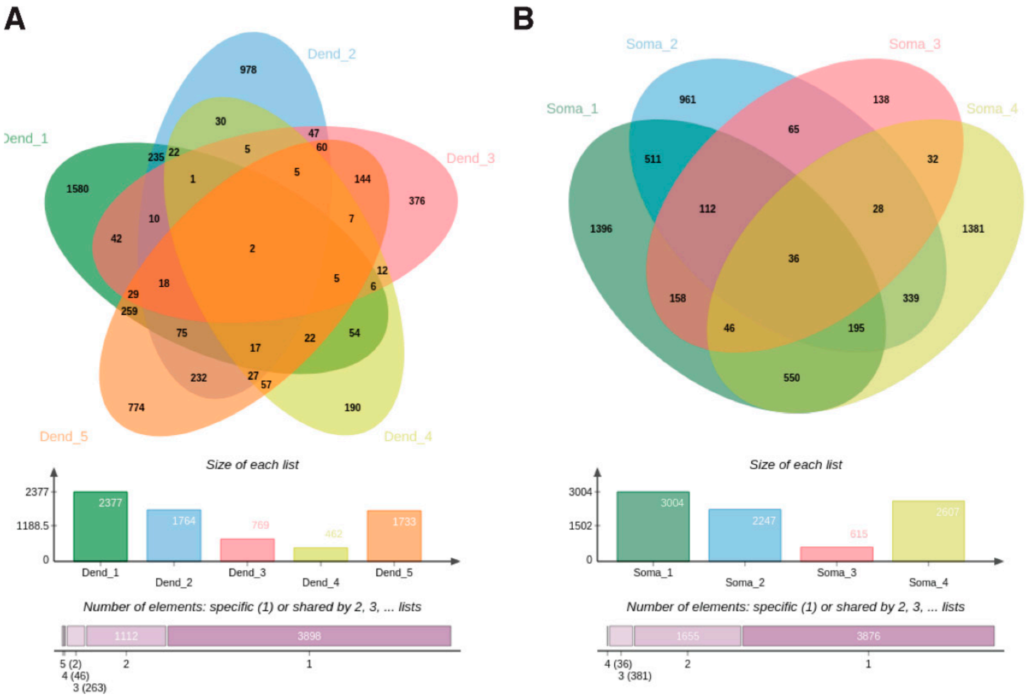

Figure S1. Venn diagrams to show the overlaps of (**A**) dendritic RNAs and (**B**) somatic RNAs across five previous studies. To collect training instances, dendritic RNAs that are overlapped in at least two studies are taken as positive instances, whereas somatic RNAs overlapped in at least two studies are taken as negative instances. The lists of dendritic RNAs from previous studies are indicated as Dend_1 [1], Dend_2 [2], Dend_3 [3], Dend_4 [4] and Dend_5 [5]. The lists of somatic RNAs include Soma_1 [1], Soma_2 [2], Soma_3 [4] and Soma_4 [5].

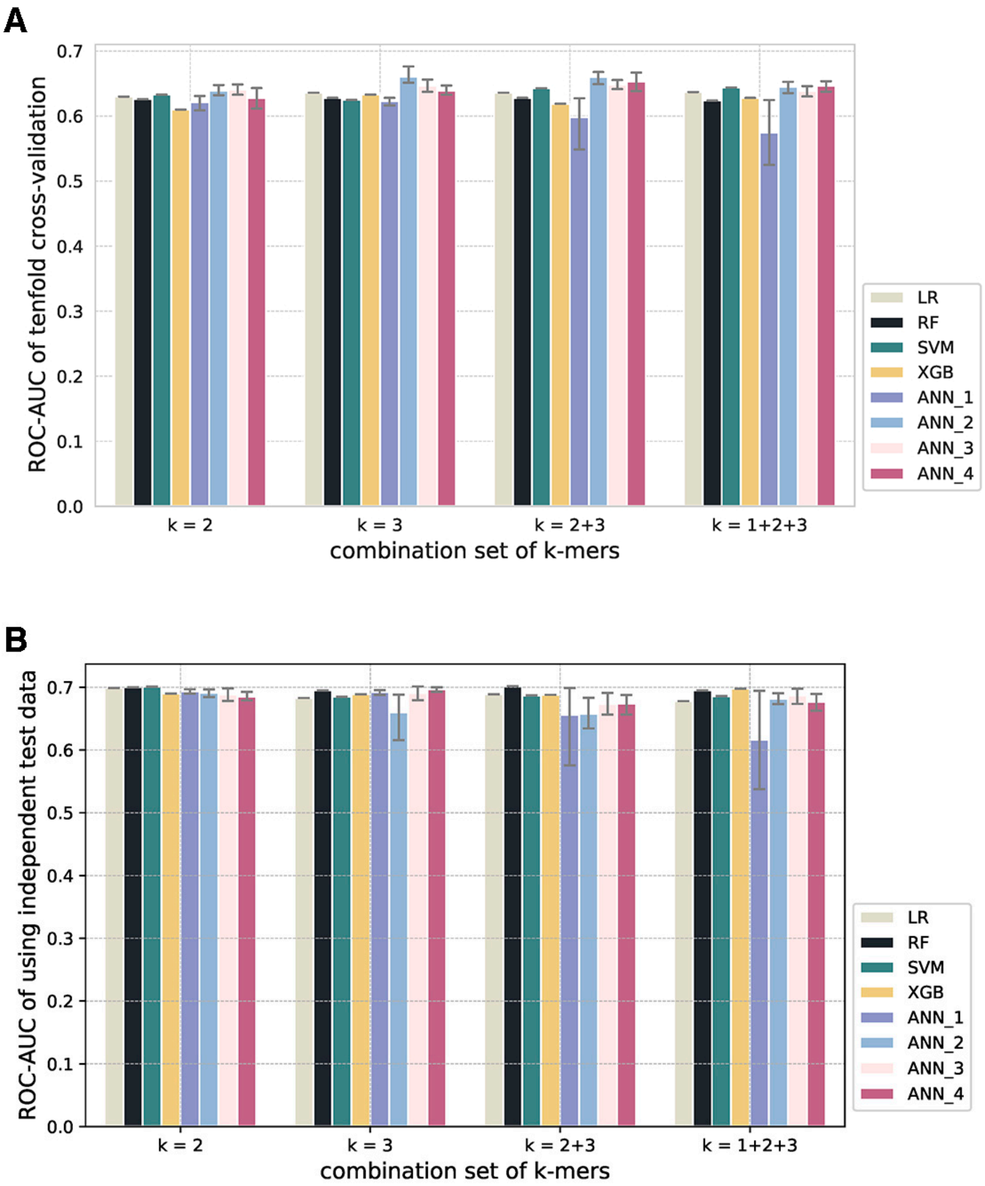

Figure S2. ROC-AUCs of the LR, SVM, RF, XGB, and ANN models with various *k*-mer features based on (**A**) five repetitions of tenfold cross-validations and (**B**) an independent test dataset. No significant difference in model performance was observed for different *k*-mer combinations. The nucleotide compositions of 1-mer, 2-mer, and 3-mer may have achieved slightly more consistent performance during tenfold cross-validations and on the independent test dataset. Different numbers (1-4) of hidden layers were tested for the ANN models.

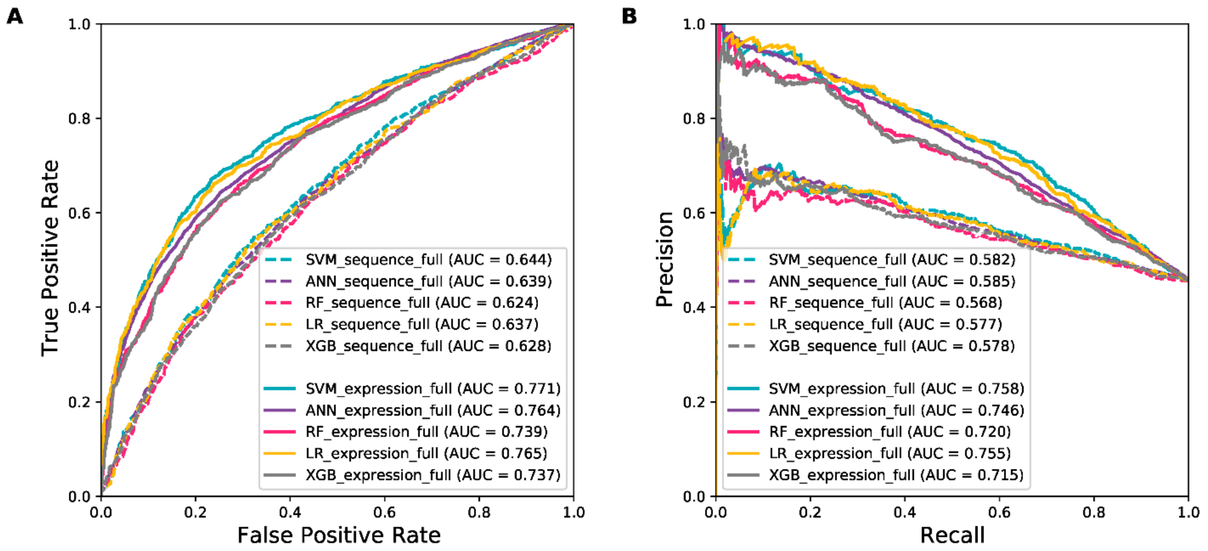

Figure S3. ROC and PR curves of the SVM, ANN, RF, LR, and XGB models with either sequence or expression features based on five repetitions of tenfold cross-validations. The area under the curve (AUC) for each model is shown in the legend.

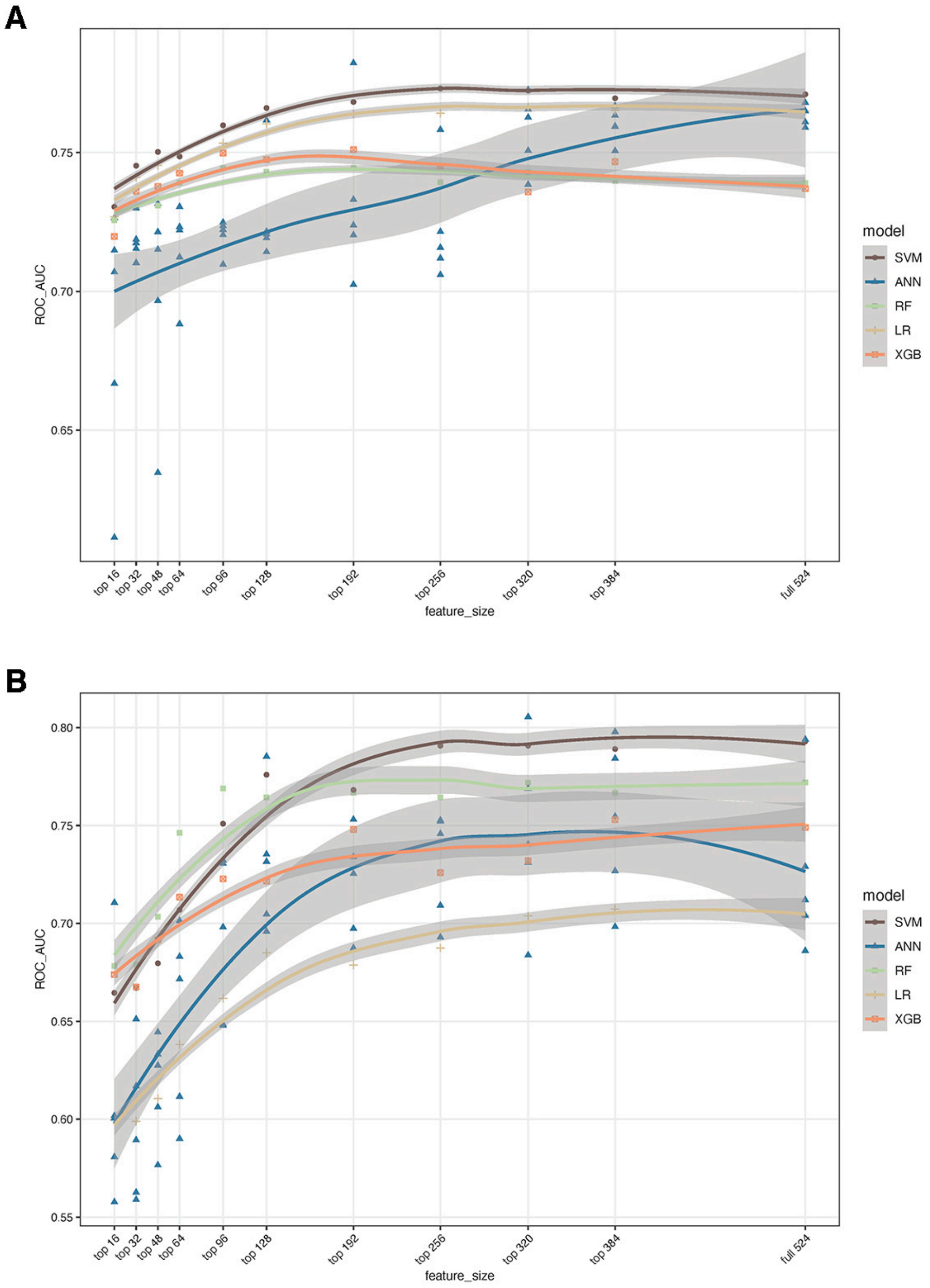

Figure S4. ROC-AUCs of the models with selected expression features based on (**A**) five repetitions of tenfold cross-validations and (**B**) the independent test dataset.

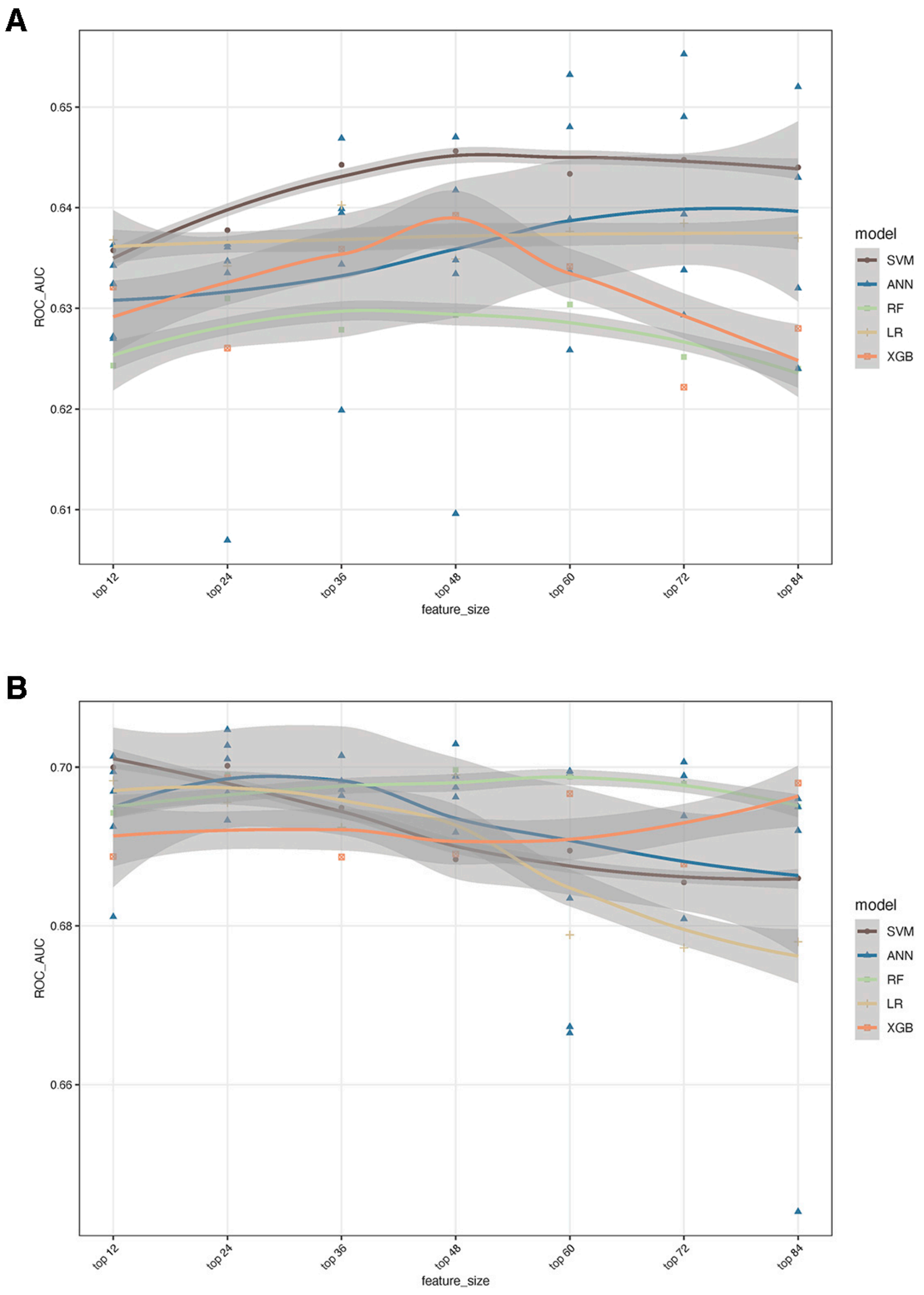

Figure S5. ROC-AUCs of the models with the selected sequence features based on (**A**) five repetitions of tenfold cross-validations and (**B**) the independent test dataset.

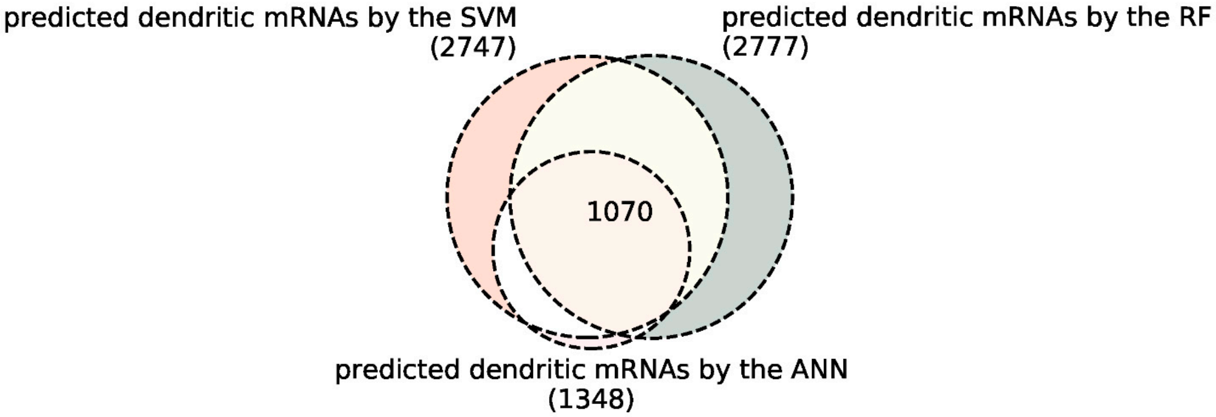

Figure S6. Venn diagram of mRNAs predicted to be synaptically localized by the SVM, ANN, and RF models using the full set of developmental brain gene expression features. The mRNAs shared by all three predictions are referred to as the high-confidence candidates for synaptic localization.

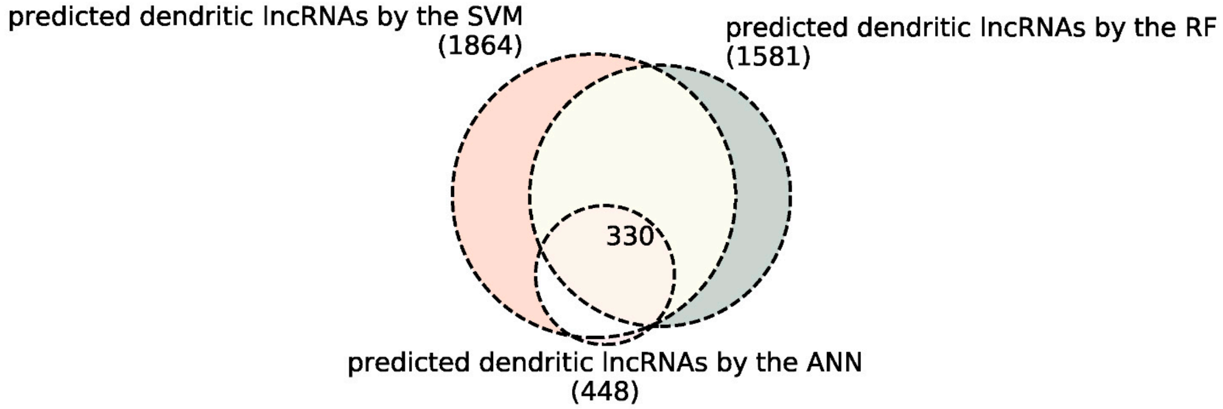

Figure S7. Venn diagram of lncRNAs predicted to be synaptically localized by the SVM, ANN, and RF models using the full set of developmental brain gene expression features. The lncRNAs shared by all three predictions are also considered to be the high-confidence candidates for synaptic localization.

Table S1. Performance comparison of artificial neural networks (ANNs) with different numbers of hidden layers based on five repetitions of tenfold cross-validations.

Features	Model	ROC-AUC	Accuracy	Sensitivity	Specificity	F1	MCC
Sequence_full	ANN_1	0.639 ± 0.011	0.603 ± 0.007	0.549 ± 0.039	0.649 ± 0.033	0.554 ± 0.021	0.201 ± 0.016
	ANN_2	0.653 ± 0.022	0.614 ± 0.016	0.553 ± 0.048	0.666 ± 0.029	0.564 ± 0.030	0.220 ± 0.035
	ANN_3	0.647 ± 0.014	0.614 ± 0.011	0.554 ± 0.026	0.664 ± 0.025	0.564 ± 0.014	0.221 ± 0.022
	ANN_4	0.655 ± 0.005	0.618 ± 0.009	0.544 ± 0.034	0.680 ± 0.041	0.558 ± 0.012	0.229 ± 0.016
Expression_full	ANN_1	0.764 ± 0.004	0.698 ± 0.010	0.649 ± 0.037	0.739 ± 0.040	0.659 ± 0.015	0.398 ± 0.014
	ANN_2	0.769 ± 0.007	0.707 ± 0.005	0.622 ± 0.027	0.778 ± 0.022	0.656 ± 0.013	0.411 ± 0.008
	ANN_3	0.761 ± 0.006	0.702 ± 0.002	0.602 ± 0.028	0.784 ± 0.024	0.645 ± 0.011	0.400 ± 0.004
	ANN_4	0.761 ± 0.023	0.702 ± 0.008	0.619 ± 0.039	0.770 ± 0.022	0.652 ± 0.018	0.403 ± 0.022

The ANNs with 1, 2, 3, or 4 dense hidden layers were tested. When using the full set of expression features, the ANNs with one and two hidden layers achieved comparable performance. To avoid model overfitting, the ANN with fewer hidden layers is preferred if model performance is comparable.

Table S2. Performance comparison of artificial neural networks (ANNs) with different numbers of hidden layers based on five repetitions using the independent test dataset.

Features	Model	ROC-AUC	Accuracy	Sensitivity	Specificity	F1	MCC
Sequence_full	ANN_1	0.684 ± 0.023	0.648 ± 0.011	0.580 ± 0.0145	0.693 ± 0.082	0.559 ± 0.083	0.274 ± 0.050
	ANN_2	0.676 ± 0.029	0.638 ± 0.032	0.623 ± 0.025	0.647 ± 0.068	0.579 ± 0.015	0.267 ± 0.048
	ANN_3	0.679 ± 0.018	0.642 ± 0.017	0.583 ± 0.078	0.681 ± 0.061	0.563 ± 0.035	0.263 ± 0.034
	ANN_4	0.659 ± 0.023	0.622 ± 0.023	0.590 ± 0.057	0.643 ± 0.064	0.554 ± 0.023	0.231 ± 0.035
Expression_full	ANN_1	0.725 ± 0.042	0.673 ± 0.041	0.493 ± 0.099	0.791 ± 0.085	0.542 ± 0.068	0.302 ± 0.081
	ANN_2	0.730 ± 0.049	0.673 ± 0.046	0.437 ± 0.129	0.830 ± 0.107	0.509 ± 0.087	0.299 ± 0.097
	ANN_3	0.736 ± 0.040	0.683 ± 0.032	0.405 ± 0.093	0.867 ± 0.040	0.500 ± 0.080	0.312 ± 0.073
	ANN_4	0.700 ± 0.078	0.682 ± 0.037	0.448 ± 0.147	0.837 ± 0.050	0.519 ± 0.100	0.312 ± 0.096

The ANNs with 1, 2, 3, or 4 dense hidden layers were tested. Using either sequence or expression features, the ANN with one hidden layer (ANN_1) achieved the best ROC-AUC.

Table S3. Training parameters tuned for the SVM, ANN, and RF models of PredSynRNA.

Model	Parameter	Parameter Description	Determined Parameter
SVM	kernel	kernel type to be used	'rbf'
	C	the penalty parameter	175
	gamma	kernel coefficient	0.001953125
ANN	batch_size	number of samples per gradient update	6
	drop_out	the dropout rate of the Dropout layer	0.01788950385827759
	hdim	hidden units of Dense layer	128
	l2_reg	L2 regularization penalty	0.00023210341057398645
	learning_rate	the learning rate of Adam optimization	0.002155448280950371
RF	n_estimators	the number of trees in the forest	45
	criterion	the function to measure the quality of a split	'entrophy'
	max_depth	the maximum depth of the tree	10
	min_samples_split	the minimum number of samples required to split an internal node	4
	max_features	the number of features to consider when looking for the best split	'sqrt'

Table S4. Performance comparison of the models with different feature sets based on five repetitions of tenfold cross-validations.

Features	Model	ROC-AUC	Accuracy	Sensitivity	Specificity	F1	MCC
Sequence_full	LR	0.637	0.605	0.582	0.625	0.573	0.206
	RF	0.624	0.597	0.523	0.660	0.542	0.184
	SVM	0.644	0.615	0.529	0.688	0.556	0.220
	XGB	0.628	0.601	0.528	0.663	0.547	0.193
	ANN	0.639	0.603	0.549	0.649	0.554	0.201`
Expression_full	LR	0.765	0.714	0.650	0.768	0.674	0.421
	RF	0.739	0.693	0.572	0.794	0.628	0.378
	SVM	0.771	0.724	0.636	0.798	0.676	0.441
	XGB	0.737	0.690	0.602	0.762	0.638	0.371
	ANN	0.764	0.698	0.649	0.739	0.659	0.398
Expression_192	LR	0.764	0.699	0.623	0.763	0.653	0.390
	RF	0.745	0.692	0.575	0.790	0.629	0.375
	SVM	0.768	0.717	0.602	0.813	0.659	0.427
	XGB	0.751	0.692	0.617	0.755	0.645	0.376
	ANN	0.732	0.674	0.645	0.698	0.643	0.346
Expression_192_sequence_12	LR	0.763	0.700	0.630	0.758	0.657	0.392
	RF	0.742	0.685	0.562	0.787	0.618	0.360
	SVM	0.771	0.723	0.633	0.798	0.675	0.439
	XGB	0.744	0.687	0.601	0.758	0.636	0.365
	ANN	0.757	0.696	0.628	0.751	0.652	0.387
Expression_192_sequence_full	LR	0.759	0.704	0.638	0.759	0.662	0.401
	RF	0.741	0.688	0.562	0.792	0.620	0.366
	SVM	0.764	0.717	0.613	0.803	0.663	0.426
	XGB	0.738	0.690	0.605	0.760	0.638	0.370
	ANN	0.754	0.693	0.600	0.771	0.639	0.381
Expression_sequence_full	LR	0.760	0.707	0.641	0.762	0.665	0.407
	RF	0.737	0.678	0.561	0.776	0.613	0.348
	SVM	0.768	0.722	0.639	0.791	0.676	0.436
	XGB	0.737	0.689	0.599	0.765	0.636	0.369
	ANN	0.769	0.701	0.648	0.744	0.661	0.399

Table S5. Performance comparison of the models with different feature sets based on five repetitions using the independent test dataset.

Features	Model	ROC-AUC	Accuracy	Sensitivity	Specificity	F1	MCC
Sequence_full	LR	0.678	0.638	0.646	0.632	0.587	0.273
	RF	0.695	0.657	0.638	0.669	0.597	0.302
	SVM	0.686	0.665	0.618	0.696	0.595	0.311
	XGB	0.698	0.666	0.639	0.684	0.604	0.319
	ANN	0.684	0.648	0.580	0.693	0.559	0.274
Expression_full	LR	0.704	0.663	0.406	0.834	0.490	0.267
	RF	0.772	0.721	0.563	0.826	0.617	0.405
	SVM	0.793	0.739	0.604	0.829	0.649	0.446
	XGB	0.749	0.704	0.545	0.809	0.594	0.367
	ANN	0.725	0.673	0.493	0.791	0.542	0.302
Expression _192	LR	0.679	0.660	0.374	0.850	0.467	0.256
	RF	0.767	0.716	0.542	0.831	0.603	0.392
	SVM	0.768	0.693	0.431	0.867	0.528	0.336
	XGB	0.748	0.698	0.573	0.782	0.602	0.361
	ANN	0.719	0.671	0.442	0.823	0.510	0.290
Expression _192_sequence_12	LR	0.691	0.659	0.396	0.832	0.481	0.256
	RF	0.769	0.718	0.548	0.830	0.608	0.397
	SVM	0.788	0.724	0.569	0.827	0.622	0.412
	XGB	0.746	0.693	0.551	0.787	0.589	0.348
	ANN	0.761	0.710	0.539	0.824	0.590	0.382
Expression _192_ sequence_full	LR	0.680	0.664	0.432	0.818	0.507	0.273
	RF	0.759	0.704	0.520	0.825	0.583	0.365
	SVM	0.757	0.698	0.483	0.840	0.560	0.349
	XGB	0.750	0.702	0.532	0.815	0.587	0.363
	ANN	0.740	0.695	0.420	0.877	0.513	0.339
Expression_sequence_full	LR	0.700	0.669	0.439	0.822	0.514	0.284
	RF	0.770	0.721	0.540	0.841	0.607	0.403
	SVM	0.780	0.715	0.588	0.800	0.622	0.396
	XGB	0.754	0.709	0.554	0.813	0.603	0.381
	ANN	0.770	0.718	0.635	0.772	0.641	0.411

References

1. Cajigas, I.; Tushev, G.; Will, T.; Dieck, S.; Fuerst, N.; Schuman, E. The Local Transcriptome in the Synaptic Neuropil Revealed by Deep Sequencing and High-Resolution Imaging. *Neuron* **2012**, *74*, 453–466, doi:10.1016/j.neuron.2012.02.036.
2. Ainsley, J.; Drane, L.; Jacobs, J.; Kittelberger, K.; Reijmers, L. Functionally Diverse Dendritic MRNAs Rapidly Associate with Ribosomes Following a Novel Experience. *Nature Communications* **2014**, *5*, 1–11, doi:10.1038/ncomms5510.
3. Taliaferro, J.M.; Vidaki, M.; Oliveira, R.; Olson, S.; Zhan, L.; Saxena, T.; Wang, E.; Graveley, B.; Gertler, F.; Swanson, M.; et al. Distal Alternative Last Exons Localize MRNAs to Neural Projections. *Molecular Cell* **2016**, *61*, 821–833, doi:10.1016/j.molcel.2016.01.020.
4. Tushev, G.; Glock, C.; Heumüller, M.; Biever, A.; Jovanovic, M.; Schuman, E. Alternative 3′ UTRs Modify the Localization, Regulatory Potential, Stability, and Plasticity of MRNAs in Neuronal Compartments. *Neuron* **2018**, *98*, 495–511, doi:10.1016/j.neuron.2018.03.030.
5. Middleton, S.; Eberwine, J.; Kim, J. Comprehensive Catalog of Dendritically Localized MRNA Isoforms from Sub-Cellular Sequencing of Single Mouse Neurons. *BMC Biology* **2019**, *17*, 1–16, doi:10.1186/s12915-019-0630-z.
